# Posterior Reversible Encephalopathy Syndrome (PRES) in a Patient with Opioid Use Disorder

**DOI:** 10.1155/2021/9999481

**Published:** 2021-06-16

**Authors:** Terence Tumenta, Samuel Adeyemo, Oluwatoyin Oladeji, Oluwole Jegede, Bordes Laurent, Tolu Olupona

**Affiliations:** ^1^Interfaith Medical Center, Department of Psychiatry and Behavioral Sciences, Brooklyn NY, USA; ^2^Yale University School of Medicine, Department of Psychiatry, New Haven, Connecticut, USA; ^3^Interfaith Medical Center, Department of Internal Medicine, Neurology, Brooklyn NY, USA

## Abstract

Posterior Reversible Encephalopathy Syndrome (PRES) is a characteristic clinical radiographic syndrome with diffuse structural alteration of cerebral white matter secondary to myelin damage with diverse and multifactorial etiologies. It can present with acutely altered mentation, somnolence or occasionally stupor, vision impairment, seizures, and sudden or chronic headaches that are not focal. The pathophysiology remains unclear, but mechanisms involving endothelial injury and dysregulation of cerebral autoregulation have been purported. We report the case of a 36-year-old male with a history of heroin use disorder, who was admitted to our hospital for opioid withdrawal. CT head without contrast and MRI with and without gadolinium showed significant white matter disease in both cerebral hemispheres and cerebellum. He was diagnosed with Posterior Reversible Encephalopathy Syndrome secondary to heroin use and managed on the medical floor in collaboration with the neurology team. His clinical symptoms improved and he was discharged after six weeks. To our knowledge, this case did not present with the risk factors for PRES reported in the literature. For patients with heroin use disorder who present with an altered mental status, PRES should be highly suspected. The diagnosis and management require collaboration between psychiatry and neurology.

## 1. Introduction

Posterior Reversible Encephalopathy Syndrome (PRES) is a characteristic clinical radiographic syndrome with diffuse structural alteration of cerebral white matter secondary to myelin damage with diverse and multifactorial etiologies [1]. PRES, also known as Reversible Posterior Leukoencephalopathy Syndrome (RPLS), can present with acutely altered mentation, somnolence or occasionally stupor, vision impairment, seizures, and sudden or chronic headaches that are not focal. PRES can unfold acutely or subacutely, with symptoms developing within hours to days [2]. Several risk factors have been described in people who are at risk for developing PRES. These risk factors include hypertension, preeclampsia, renal failure, kidney diseases causing secondary hypertension and hypovolemia, liver disease, exposure to cytotoxic or immunosuppressant medications, autoimmune disorders, and sepsis. Patients with PRES often have one or more of these risk factors [2]. The pathophysiology of PRES remains unclear, but some have proposed that the mechanisms center around endothelial injury and dysregulation of cerebral autoregulation, which is the brain's ability to maintain constant cerebral blood flow over a range of blood pressures through constriction or dilation of cerebral blood vessels [2–4]. Headaches, seizures, focal neurological deficits, visual changes, nausea, or vomiting, and altered mental status are frequently associated with PRES. The features of PRES seen on MRI are primarily characterized by vasogenic edema in central areas, often followed by typical regions. When recognized promptly, most cases can be reversed in 2-3 weeks with proper treatment [3]. PRES has also been reported in children [5–10] and pregnant women with eclampsia and preeclampsia [10].

Clinical and radiographic syndromes that overlap with PRES have been described in overdoses of drugs of abuse like benzodiazepines, amphetamines, and opiates. Drugs of abuse increase blood-brain barrier permeability, which, in turn, increases the influx of peripheral toxins into the brain [11, 12]. Protein disruption at tight junctions, neuroinflammation, oxidative stress, and production of reactive oxygen species have all been postulated as fundamental mechanisms through which drugs alter the blood-brain barrier structure and integrity. Heroin's effects indirectly involve its metabolites (morphine and 6-MAM) that act as substrates in P-glycoprotein membrane regulation. P-glycoprotein inhibition at the blood-brain barrier acutely disrupts the blood-brain barrier permeability and selectivity in the nucleus accumbens [11, 12]. Cases of toxic leukoencephalopathy have been reported in patients with illicit heroin vapor inhalation [13–16]. However, literature about heroin use and PRES is scarce. We hereby report the case of a 36-year-old- male, who was admitted to our hospital for opioid withdrawal. He subsequently deteriorated and was diagnosed with PRES. This case is unique in that the patient did not present with the classic risk factors and features associated with PRES and to our knowledge, not many cases of PRES have been linked to heroin use. This report shows that PRES is a possible occurrence in patients who use heroin. The diagnosis of PRES is made by the clinical history, neurologic examination, and neuroimaging. This requires a high index of suspicion because diagnosis is the initial step in treatment. Though reversible, severe cases of complications have been reported that are not reversible and can have permanent neurologic damage.

## 2. Case Report

A 36-year-old man, domiciled with his wife, employed as a manager and part owner at a restaurant, presented to the psychiatry emergency room for psychiatric evaluation. The patient was accompanied by his wife and had no significant past medical or psychiatric history. During the initial evaluation, the patient appeared disoriented, agitated, restless, and was unable to participate in the interview. He presented with a flushed face, runny nose, goose flesh, pinpoint-sized pupils, and a resting pulse rate of 104 bpm. He was noted to have mild hand tremors, rubbed his joints constantly, and yawned incessantly. He denied gastrointestinal symptoms and denied substance use.

The patient's wife reported that the patient had been taking medications for shoulder pain sustained at his job. She could not, however, provide any further details but reported that six months prior to presentation, the patient's behavior had been “weird,” as he would fall asleep unexpectedly even when driving. Collateral information was obtained from the patient's Primary Care Physician (PCP) who reported that the patient had an addiction to heroin and had recently been started on methadone maintenance therapy five months prior to presentation. The patient was on methadone 40 mg po daily at the time of presentation. According to the PCP, despite being prescribed methadone, the patient was noncompliant and continued to use heroin. However, he did not provide details of the patient's heroin use pattern.

Urine toxicology screen was positive for opiates and negative for methadone. His Clinical Opiate Withdrawal Scale (COWS) score at presentation was 19/47, indicating moderate opioid withdrawal.

**Figure 1:**
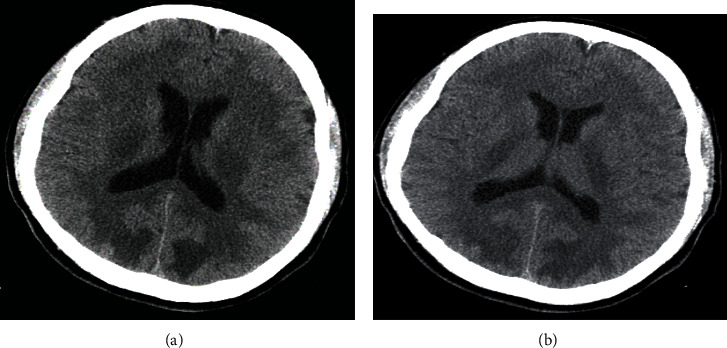
(a, b) Computed tomography scan of the brain without contrast, showing white matter disease in both cerebral hemispheres and cerebellum.

**Figure 2:**
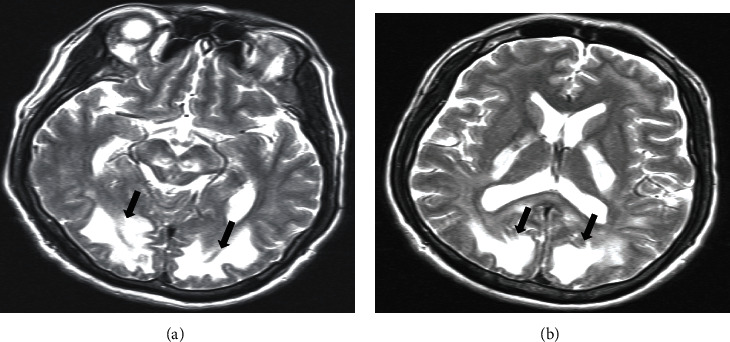
(a, b) T2-weighted Magnetic Resonance Imaging of the brain, showing abnormal signal changes in the periventricular white matter, severe in occipital lobe regions, with mass effect on pons and medulla.

Due to his altered mentation, the patient was admitted to the medical floor and started on methadone tapering protocol over three days. The neurology team was consulted for persistently altered mentation, and they evoked a possible damage to the blood-brain barrier from chronic heroin abuse versus hypoxic encephalopathy (probable hypoxia in the field secondary to respiratory depression from heroin toxicity). Although the patient's oxygen saturation was normal on admission, he had three episodes of hypoxia (the lowest saturation was 92%) within the first three days of admission. Imaging studies provided further insight into the patient's condition. The computed tomography scan of the head without contrast showed physiologic calcifications within the pineal and choroid plexus and marked and significant abnormalities involving the white matter, diffusely, throughout the right and left cerebral hemispheres and cerebellum (Figures 1(a) and 1(b)). Magnetic Resonance Imaging of the brain done five days later revealed that the constellation and pattern of findings were highly suggestive of posterior reversible encephalopathy syndrome (Figures 2(a) and 2(b)). Looking at the patient's clinical history, neurologic examination findings, and neuroimaging results, the diagnosis of PRES was affirmed.

Differential diagnostic considerations included status epilepticus, hypoglycemia, acute cerebral ischemic infarction, acute hypertensive encephalopathy, and toxic leukoencephalopathy. Hypoglycemia was ruled out as there was no evidence from history and laboratory investigations to support the diagnosis. Acute hypertensive encephalopathy was ruled out due to lack of history and the patient's blood pressure and kidney function trends during his hospitalization. Although the patient had a few blood pressure spikes, it was within normal range and his mean arterial blood pressure remained stable as well as his kidney function. A viral or autoimmune origin was ruled out as immunology, and serology results were all within normal limits. Lumbar puncture showed no significant findings. EEG could not be performed due to a lack of availability in our hospital. Toxic leukoencephalopathy was also considered, given the similarity of clinical presentations and difficulties to distinguish PRES from toxic leukoencephalopathy. However, imaging studies were more suggestive of PRES.

The patient was managed with low-dose antihypertensives to avoid blood pressure spikes and with corticosteroids to reduce cerebral edema. Given the altered mentation, white matter changes, and high risk for seizures, levetiracetam was given prophylactically. He also received physical therapy to prevent deconditioning. The patient had a slow clinical recovery over 6 weeks and was discharged to a subacute rehabilitation facility, with outpatient follow-up with the neurology team.

## 3. Discussion

There have been several case reports about Posterior Reversible Encephalopathy Syndrome (PRES), also known as Toxic Leukoencephalopathy resulting from methadone toxicity and/or heroin overdose [8, 14, 17]. Heroin has lipophilic properties and acetylation of its hydroxyl groups increases its blood-brain barrier penetration by 100-fold [11]. It has a high affinity for mu receptors which are densely populated in the cerebellum and limbic system in humans as supported by postmortem studies [18]. The exact mechanism by which heroin abuse results in damage to the white matter is not fully understood; however, endothelial dysfunction, hypoxia from respiratory depression, and autoregulatory failure are the plausible proposed pathophysiological basis. In this case presentation, there was no report of significant past medical history pointing to other risk factors or etiologies for PRES; hence, a long-standing history of opioid use disorder likely exposed him to chronic insults from damage to the blood-brain barrier and hypoxic injury from periods of an apparent overdose. His presentation with altered mental status in the context of opiate withdrawal was atypical as most reported cases of PRES or toxic leukoencephalopathy were associated with opioid intoxication and not withdrawal [14, 17, 18]. As a clinical radiographic syndrome, a diagnosis of toxic leukoencephalopathy cannot be made without corroborating neuroradiologic evidence and a normal finding on MRI would suggest systemic disorders like uremia, hypothyroidism, or hepatic encephalopathy [19]. In this case presentation, there was no evidence suggestive of infection, trauma, metabolic disorder, demyelinating disease, or exposure to other toxins that may have resulted in PRES or toxic leukoencephalopathy. Although the patient had a few blood pressure spikes, it was within normal range and his mean arterial blood pressure remained stable, as well as his kidney function. The patient's oxygen saturation was normal on admission; however, it is worthy to note that within the first three days on admission, our patient experienced three episodes of hypoxia (the lowest saturation was 92%). The diagnosis of PRES was considered after 5 days of treatment after excluding other possible pathologies. A diagnosis of PRES requires a high index of suspicion and early recognition is crucial for prompt management [3]. Altered mental status, deficits in memory and attention, headache, seizures, confusion, visual disturbances, focal neurological deficits, and ataxia are common features of PRES [7, 19]. In our case, confusion, altered mental status, and heroin withdrawal were prominent symptoms. Weber et al. proposed that clinicians should have a heightened suspicion for PRES or toxic leukoencephalopathy, when confronted with a constellation of signs and symptoms in the context of heroin use [20]. These include heroin withdrawal, cerebellar disease, and symmetrical white matter lesions affecting the cerebrum, cerebellum, and cerebellar peduncles [17, 20]. As shown in Figure 2, MRI findings in our case were highly compatible with radiologic features of PRES, which include diffuse hyperintensity of cerebral white matter and involvement of the cerebellum [21, 22]. T-2-weighted MRI is preferred because of its superior ability to display lesions of the white matter [23]. It is important to differentiate PRES from toxic leukoencephalopathy. Both PRES and toxic leukoencephalopathy syndromes have been described as potentially reversible. They have similar clinical presentations but have characteristic MRI appearances that differ. While toxic leukoencephalopathy is potentially reversible like PRES, a higher morbidity and mortality has been associated with it [16]. There have been reports of simultaneous occurrence of both syndromes [24], while other studies have also reported on the difficulties to distinguish PRES from toxic leukoencephalopathy [25].

Treatment of PRES involves the removal of the offending agent and supportive management. The use of antioxidants such as ubiquinone (Coenzyme Q) and high doses of Vitamins C and E have been reported to aid and improve the resolution of clinical and radiological syndromes [26, 27]. However, this treatment was reported for a few case reports, and there is no specific therapeutic strategy that is currently available [28, 29]. Other treatment measures include the use of glucocorticoids, management of blood pressure changes, and monitoring for life-threatening complications like coma. No randomized trials have been conducted for various treatments used [28]. A high suspicion for severe cerebellar edema is warranted in cases of severe opioid toxicity that present with a dramatic decline in the level of consciousness during the acute withdrawal phase. This situation may require admission to the ICU for management of raised intracranial pressure [18, 28]. The prognosis of PRES is believed to correlate with the severity and anatomical extent of white matter damage and ensuing complications, with most cases undergoing complete resolution and severe cases resulting in persistent clinical deficits or mortality.

## 4. Conclusion

There has been a recent increase in reports of PRES or toxic encephalopathy in patients with heroin use disorder. The diagnosis requires a high index of suspicion as well as an astute clinical examination with adequate imaging procedures. Although this condition appears reversible, and treatment is readily available, a delay in the diagnosis could have deleterious consequences for the patient given the disruption of the blood-brain barrier. A collaborative care model between psychiatry and neurology is imperative in the diagnosis and management of this condition.

## Data Availability

Any questions should be addressed to the corresponding author about the supporting data availability.
